# Incident characterization of health conditions in women suspected of being victims of intimate partner violence

**DOI:** 10.1371/journal.pone.0326235

**Published:** 2025-07-16

**Authors:** Mireille Sam, Joana Barrocas, Maria Clemente-Teixeira, Tiago Taveira-Gomes, Maria João Vidal-Alves, Teresa Magalhães

**Affiliations:** 1 Department of Public Health and Forensic Sciences, and Medical Education, Faculty of Medicine, University of Porto, Porto, Portugal; 2 USF Caravela, Local Healthcare Unit of Matosinhos, Lagoa Street, Senhora da Hora, Portugal; 3 Abel Salazar Biomedical Sciences Institute, University of Porto, Porto, Portugal; 4 Center for Health Technology and Services Research (CINTESIS), Porto, Portugal; 5 MTG Research and Development Lab, Porto, Portugal; 6 Department of Community Medicine, Information and Decision in Health, Faculty of Medicine, University of Porto, Porto, Portugal; 7 Faculty of Health Sciences, University Fernando Pessoa (FCS-UFP), Porto, Portugal; 8 Epidemiology Unit (EPIUnit), Laboratory for Integrative and Translational Research in Population Health (ITR), Porto, Portugal; 9 TOXRUN–Toxicology Research Unit, University Institute of Health Sciences, Advanced Polytechnic and University Cooperative (CESPU), CRL, Gandra, Portugal; Edo State University Uzairue, NIGERIA

## Abstract

Intimate partner violence (IPV) is a serious public health problem due to its severe health outcomes and its association with significant morbidity and mortality. There are many prevalence studies about this topic but few on incidence. Therefore, an incident health characterization of alleged IPV victims may be relevant to understanding the situation of the person at the moment when the suspicion of violence was recorded. The global aim is to characterize the health conditions of women allegedly victims of IPV. Specifically, the study aims to characterize: (a) health risk behaviours; (b) traumatic injuries and intoxications; (c) mental health disorders; and (d) physical disorders. We conducted a retrospective cohort study based on secondary data analysis of electronic health records and health registry data of patients of the Local Health Unit of Matosinhos (Portugal) between 2001 and 2021. To analyse variables, we selected official codes and regular terms used by physicians to describe violence during any clinical encounter. A total of 16 966 alleged female victims of IPV were identified along with a matched population of 29 980 women. This study highlights the significant increase in incidence rates of various health conditions among those allegedly victimized compared to the matched population. It provides novel insights by comparing incidence rates with previous prevalence estimates, revealing higher incidence ratios across the majority of health conditions among allegedly female IPV victims. This suggests a potential loss of follow-up among these individuals, possibly due to treatment dropout or mortality, highlighting the urgent need for targeted interventions and enhanced healthcare support for IPV victims.

## Introduction

In 1979, the United Nations (UN) issued its first *Convention on the Elimination of Discrimination Against Women*, marking a pivotal step in the UN’s pursuit of sex equality. Despite this, nowadays, violence against women continues to be a frequent and severe violation of human rights, namely in the setting of intimate partner relationships. [1–3]

Intimate partner violence (IPV) is characterized, among others, by being repeated. [4,5] Approximately 50–60% of women who experienced it, reported enduring multiple episodes of victimization by the same partner, with these incidents lasting a year or more. [6]

Evidence shows that IPV against women can lead to both fatal and non-fatal consequences (in the physical, emotional, and social domains). These consequences can manifest as acute or chronic health conditions. [7,8]

Physical health short-term conditions of IPV are better known and include acute traumatic injuries and acute psychological trauma. [7,8]

Long-term health outcomes may result from chronic stress linked to repeated violent episodes and can persist long after the violence has ended, increasing inflammation and cortisol levels, which may be associated with multiple health disorders. [9–12] For example, violence may be connected with cardio and cerebrovascular risk, causing higher report rates of dyslipidemia, hypertension, heart disease (4.2% in victims vs 3.0% in non-victims) and stroke (3.2% in victims vs 2.0% in non-victims). [13,14] Regarding women’s sexual and reproductive health, IPV victims are 3 times more likely to have gynaecological problems than the average woman, with a 1.5 times higher likelihood of having sexually transmitted infections, including HIV. [9,15] They are also at a higher risk of unwanted pregnancies and induced abortion. [16] Exposure to violence during pregnancy increases the likelihood of antepartum haemorrhage, intrauterine growth restriction, miscarriage (1.2 times more likely), preterm labour (1.4 times more likely), premature rupture of membranes (3.2 times more likely) and a higher tendency to delay prenatal care (2.6 times) than other mothers. [15,17–20] They also present more mental health disorders, specifically sleep disorders, unspecified chronic pain, anxiety disorders and major psychiatric disorders, being 3.6, 2.4, 2.7 and 2.2 times higher than in the general population, respectively. Suicidal ideation is also higher (8.6 times more prevalent). [8] Furthermore, exposure to violence escalates health risk behaviours, with research showing an increased prevalence of subsequent tobacco use, alcohol abuse and illicit drug use (1.5, 6.5, and 13 times higher, respectively). [8] In addition, female victims are more likely to have a poorer diet and greater medication consumption (specifically anxiolytics, sedatives, antidepressants, and antipsychotics: 1.7, 2.0, 2.1, and 3.2 times greater, respectively) than the general population. [8,15,21]

These alarming findings emphasize the urgent need for collective action to address IPV as a serious public health problem, as it is recognized by the World Health Organization. [2,22]

Nevertheless, while most studies conducted in this field focus on characterizing suspected victims of violence and estimating its burden, namely regarding its health consequences, scarce studies have been conducted concerning the temporal dynamics of this phenomenon.

Considering prevalence, studies about violence health outcomes, directed by our team, offer us the burden of the health problems that may be associated with violence, focusing on a patient population with regular follow-up on primary care. [8,10,23] But the prevalence estimates we found are considerably lower than reported by other kinds of studies about domestic violence (DV) in general. [23,24] This leads us to hypothesize that a substantial number of victims may abandon regular care and disconnect from the health system care when they feel they have been identified as such. In fact, the victims’ decision not to leave the abusive relationship, abandoning any institutional intervention, is a very common situation in these cases. [25,26] However, it is well known that the precocity of violence detection and the precocity and quality of professional intervention (mainly treatment and protection), are fundamental to stop revictimization and reduce, or even avoid, its health outcomes. [27–29]

Thus, an incident health characterization of the IPV allegedly victims’ population may be relevant to understanding the situation of the person at the moment where the register of the violence suspicion has been made (this is, the closer possible to the real moment of violence).

Moreover, evidence indicates that the severity of violence is associated with a higher use of healthcare services. [12] Hospitals, particularly the emergency department, and primary health centres act, frequently, as the primary entry points of these cases to the health care system, playing a crucial role in the identification and management of violence, by providing referrals and treatment for both violence and its associated consequences. [22] These institutions can ensure continuous support for victims and survivors by connecting them with necessary support services and helping in the promotion of their overall health and well-being. [30]

In the Portuguese context, a local healthcare unit (Unidade Local de Saúde – ULS) of the National Health Service (NHS) includes an emergency department and primary healthcare facilities. Thus, it serves as a relevant source to investigate IPV cases against women within specific geographic areas. [7]

Our study is carried out under comparable conditions and within the same population as a previous prevalence study at the ULS of Matosinhos (ULSM). [8] The general aim is to characterize the health conditions of women, at the time of medical recording of clinical elements compatible with being suspected of IPV victimization. Specifically, we will characterize (a) health risk behaviours; (b) traumatic injuries and intoxications; (c) mental health disorders; and (d) physical disorders.

## Materials and methods

### Materials

We conducted a retrospective cohort study. It was based on secondary data analysis of electronic health records (EHR) and health registry data of patients of the ULSM, a northern Portuguese centre that provides primary, secondary, and tertiary health care to an urban population of approximately 176 672 inhabitants, throughout 14 primary care centres and 1 hospital. Furthermore, the ULSM receives patients from other locations and institutions to provide specific care.

The inclusion criteria were: (a) women; (b) aged 16–60 years old; (c) who had visited the ULSM between 1 January 2001 and 31 December 2021; (d) presenting documented suspicion of being a victim of IPV in clinical notes; (e) with at least one clinical record entry in the 365 days before criteria (d). We found 16,966 women to be characterized.

For comparison analysis, we defined a cohort of women observed at median age found in the IPV group (age-matched population – n = 29 980).

Data access for analysis was granted after approval by the ULSM *Health Ethics Committee* and data protection officer.

### Methods

We consider IPV as the presence of, at least, one of the official codes or regular expressions listed and used in a past publication on the same database [8]. The clinical notes (regular expressions and codes) related to IPV, could have been documented by any healthcare professional during any clinical encounter, whether in primary healthcare or hospital settings. The expressions were selected based on the most frequently used terms to describe IPV situations within the clinical setting of health services. Codes were selected using the International Classification of Diseases (ICD-9 and ICD-10), the International Classification of Primary Care (ICPC-2), and the Anatomical Therapeutic Chemical Classification System (ATC).

The index visit was defined as the appointment during which the healthcare provider recorded the woman as a suspected victim of IPV in the clinical registry data.

We analyzed the following variables: (a) age; (b) social conditions; (c) health risk behaviors; (d) traumatic injuries and intoxications; (e) mental health disorders; and (f) physical disorders.

All data processing and analysis were exclusively performed by analytical programs specifically developed for this purpose and sent for execution on ULSM servers. Data extraction took place in march 2023 and did not occur beyond the ULSM and no direct access by the researchers took place. To enhance the security level, and per the Health Insurance Portability and Accountability Act (HIPAA), the processed data were de-identified by the ULSM *Information Technology Department* before analytic code execution.

A descriptive analysis of relative and absolute frequencies for all variables was carried out, without other statistical studies. The percentage of null values (ø) was calculated for all variables that were computed with information aside from diagnosis codes (data not shown). The ratio of the assessed rates of the health conditions between both populations (suspected/matched) was used to highlight the magnitude of the increase in the rates for the alleged victims when compared to the matched population.

## Results

The median age of women allegedly victims of IPV was 41 years (Table 1).

**Table 1:** Age distribution of alleged female victims (n = 16 966).

Age groups (years)	Females – n (%)
16-20	962 (5.7)
20-30	2 784 (16.4)
30-40	4 124 (24.3)
40-50	4 352 (25.7)
50-60	4 340 (25.6)

Some of their social conditions are presented in Table 2.

**Table 2:** Social conditions – n (%).

	Suspected population (n = 16 966)	Matched population (n = 29 980)	Ratio
Poverty	133 (0.8)	47 (0.2)	4.0
Unspecified social problem	49 (0.3)	25 (0.1)	3.0

Tables 3–6 show the comparison between health conditions in both populations.

**Table 3:** Health risk behaviours – n (%).

	Suspected population (n = 16 966)	Matched population (n = 29 980)	Ratio
Alcohol abuse	81 (0.5)	21 (0.1)	5.0
Drug abuse	13 (0.1)	5 (0.02)	5.0
Current smoking	3 355 (19.8)	3 213 (10.7)	1.9

**Table 4:** Traumatic injuries and intoxications – n (%).

	Suspected population (n = 16 966)	Matched population (n = 29 980)	Ratio
Dislocations	498 (2.9)	277 (0.9)	3.2
Crushing injuries	121 (0.7)	75 (0.3)	2.3
Burns	598 (3.5)	386 (1.3)	2.7
Open wounds	4 599 (27.1)	3 956 (13.2)	2.1
Fractures	6 148 (36.2)	6 165 (20.6)	1.8
Superficial injuries	489 (2.9)	493 (1.6)	1.8
Poisoning	2 477 (14.6)	2 440 (8.1)	1.8

**Table 5:** Mental health disorders – n (%).

	Suspected population (n = 16 966)	Matched population (n = 29 980)	Ratio
Suicidal ideation	87 (0.5)	25 (0.1)	5.0
Social deprivation	267 (1.6)	120 (0.4)	4.0
Memory disorders	67 (0.4)	29 (0.1)	4.0
Sleep disorders	1 249 (7.4)	728 (2.4)	3.1
Unspecified chronic pain	1 581 (9.3)	945 (3.2)	2.9
Psychosocial stress	3 925 (23.1)	2 639 (8.8)	2.6
Major psychiatric disorders	7 507 (44.3)	5 192 (17.3)	2.6
Headache	363 (2.1)	261 (0.9)	2.3
Unspecified illness	274 (1.6)	202 (0.7)	2.3
Eating disorders	34 (0.2)	24 (0.1)	2.0
Post-traumatic stress disorder	26 (0.2)	16 (0.1)	2.0
**Medication**			
Antipsychotics	2 074 (12.2)	1 389 (4.6)	2.7
Sedatives	5 711 (33.7)	4 299 (14.3)	2.4
Antidepressants	9 007 (53.1)	6 894 (23.0)	2.3
Anxiolytics	11 175 (65.9)	9 816 (32.7)	2.0

**Table 6:** Physical disorders – n (%).

	Suspected population (n = 16 966)	Matched population (n = 29 980)	Ratio
Type 2 diabetes	1 053 (6.2)	487 (1.6)	3.9
Non-alcoholic fatty liver disease	124 (0.7)	46 (0.2)	3.5
Obesity	3 467 (20.4)	2 757 (9.2)	2.2
Hypercholesterolemia	7 105 (41.9)	6 716 (22.4)	1.9
Metabolic syndrome	11 453 (67.5)	11 910 (39.7)	1.7
Hemorrhagic stroke	36 (0.2)	8 (0.03)	6.7
Early heart disease	104 (0.6)	22 (0.1)	6.0
Chronic kidney disease	170 (1.0)	48 (0.2)	5.0
Myocardial infarction	90 (0.5)	20 (0.1)	5.0
Ischemic stroke	309 (1.8)	122 (0.4)	4.5
Hypertension	3 664 (21.6)	2 090 (7.0)	3.1
Sexually transmitted infections	224 (1.3)	186 (0.6)	2.2
Voluntary abortion	170 (1.0)	182 (0.6)	1.7
Natural Abortion	381 (2.3)	626 (2.1)	1.1
Preeclampsia	125 (0.7)	214 (0.7)	1.0
Chronic obstructive pulmonary disease	103 (0.6)	25 (0.1)	6.0
Asthma	1 151 (6.8)	946 (3.2)	2.1
Urinary tract infection	571 (3.4)	602 (2.0)	1.7
Inflammatory pelvic disease	52 (0.3)	52 (0.2)	1.5
Chronic immune-inflammatory disorder	276 (1.6)	232 (0.8)	2.0
Cervical cancer	85 (0.5)	61 (0.2)	2.5
Cancer (in general)	2 074 (12.2)	1 568 (5.2)	2.3

## Discussion

The results of this study highlight the increased incidence rates of health conditions in women allegedly victims of IPV compared to the matched population. We observed that poverty rates are 4 times higher among women who are allegedly victims of IPV, which aligns with previous studies showing that poverty is an important risk factor for IPV [31–33], even though this is a problem that cuts across all societies and transcends socio-economic boundaries, affecting individuals across various income levels. [34–38] The most frequent health conditions observed were metabolic syndrome (67.5%), anxiolytic consumption (65.9%), antidepressant consumption (53.1%), and major psychiatric disorders (44.3%) – Tables 5 and 6. The highest ratios were observed for hemorrhagic stroke (6.7 times higher compared to the matched population), followed by chronic obstructive pulmonary disease and early heart disease (both 6 times higher) – Table 6. This indicates that among all studied health conditions, these 3 outcomes stand out as having the highest occurrences in the suspected population when compared to the matched population.

The study examined the same population, under similar conditions, as a previous prevalence study conducted by our team at the ULSM. [8] This earlier study found that prevalence estimates were considerably lower than reported in other literature. However, in the current study, we observed quite the opposite, identifying very high incidence rates of health conditions among women medically recorded as alleged victims of IPV, which raises concern and requires deep analysis.

### Health risk behaviours

As described in Table 3, alleged female victims are 5 times more likely to have alcohol and drug abuse problems, and 1.9 times more likely to be current smokers, when compared to the matched population. These results are consistent with previous studies that suggest that victims may resort to engaging in high-risk behaviours, including smoking, heavy drinking, drug abuse, and sexual risk behaviours (e.g., multiple partners, unprotected intercourse), as a means of coping with the physical and psychological pain associated with traumatic situations. [13,19,39–42] Other risk behaviours that can be observed in victims of IPV include physical inactivity, greater use of medications and poor diet. [43]

### Traumatic injuries and intoxications

IPV is a significant contributor to physical injuries inflicted by others, including fractures, lacerations, contusions and frequently, damage to the head, neck or face. [44,45] It is also strongly linked with accidental injuries and self-harm behaviours as an expression of emotional distress underscoring the profound impact of such violence on mental health. [12,46]

We identified higher rates of traumatic injuries and intoxications in alleged IPV victims as can be seen in Table 4. The rates of dislocations and crushing injuries are 3.2 and 2.3 times higher than the matched population, respectively. The same applies, in descending order, to other traumatic outcomes and intoxications: burns (2.7 times higher), open wounds (2.1 times higher), fractures, superficial injuries and poisoning (1.8 times higher in all three conditions). Although it is known that superficial injuries are the most frequent in IPV cases, the most common injuries reported were fractures (36.2%) and open wounds (27.1%). [47] This discrepancy may be attributed to the increased attention given to more severe injuries. However, it is crucial to emphasize the significance of valuing less serious and superficial injuries, despite their seemingly minor nature, as they can serve as important indicators of IPV. [7,48]

### Mental health disorders

IPV significantly affects victims’ mental health, leading to emotional distress and contributing to the morbidity and mortality associated with IPV. Social isolation can elevate the risk of both experiencing IPV and encountering negative health outcomes, contributing to the overall health burden experienced by affected individuals. [13] Female victims of IPV are more likely to experience a range of mental health challenges, such as major depression and depressive symptoms, anxiety disorders (including panic attacks), post-traumatic stress disorder (PTSD), sleep and eating disorders, social dysfunction/isolation, cognitive impairments (memory loss and dizziness), psychotropic drugs use, suicidal behaviour (suicidal ideation and attempts), and chronic pain (including headaches). [24,49–53] Research indicates that physical violence has a more pronounced impact on mental health outcomes compared to psychological abuse, doubling the risk of poorer mental health among abused women compared to those who have not reported IPV. [54] Furthermore, abuse of women is linked to somatization, where factors such as the perception of abuse by the victim play a significant role in its development. Healthcare providers must consider abuse when encountering women with somatization symptoms. [55]

Contrary to previous research findings, our study reports a lower incidence of suicidal ideation among alleged cases of IPV, with only 0.5% of cases identified. However, it’s worth noting that suicidal ideation is still 5 times more likely compared to the matched population. [24] Regarding mental health disorders, our findings align with previous research. Sleep disorders and chronic pain were found to be 3.1, and 2.9 times more likely in the suspected cohort, respectively. Social deprivation and memory disorders exhibited a fourfold increase compared to the matched population, while psychosocial stress and major psychiatric disorders were 2.6 times more likely to occur. The incidence of headaches and unspecified illnesses was 2.3 times greater, and eating and post-traumatic stress disorders were twice as likely among alleged victims of IPV. [56]

The likelihood of psychotropic drug use was significantly higher, with antipsychotics being 2.7 times more incident, sedatives 2.4 times, antidepressants 2.3 times, and anxiolytics twice as high. Notably, anxiolytics were the most frequently used class of drugs, accounting for 65.9% of cases.

### Physical disorders

In addition to the immediate and direct consequences, IPV significantly impacts victims’ long-term health. [44] IPV is often characterized by a chronic and recurrent nature, leading to sustained exposure to stress. Over time, this chronic stress shapes the neurobiological response of victims, resulting in changes at both central and peripheral levels that correlate with genetic factors and phenotypes of non-communicable diseases. Individuals who have experienced IPV throughout their lifetime are at higher risk of developing a range of chronic diseases. These may include metabolic disorders such as hypercholesterolemia and diabetes, cardiovascular diseases like hypertension and myocardial infarction, cerebrovascular diseases including stroke, respiratory disorders such as chronic obstructive pulmonary disease (COPD), inflammatory diseases like joint disease and asthma, neoplastic diseases, and activity limitations. [10,13,57–59]

Furthermore, IPV is strongly linked to the sexual and reproductive health of victims. Previous research highlights that IPV victims are less likely to report condom use at their last vaginal intercourse and are approximately twice as likely to believe their partner has concurrent partners. Given that partner concurrency is a known risk factor for contracting sexually transmitted infections (STIs), this poses a significant health risk to IPV victims. [60,61] Additionally, victims not only face a higher risk of unintended pregnancies but also an increased likelihood of miscarriage. [62]

In the area of general health conditions, suspects were more likely to have metabolic syndrome (67.5%), hypercholesterolemia (41.9%) and hypertension (21.6%) – Table 6. Consistent with previous research, we found that all physical disorders analysed were more incidents in alleged victims of IPV than in the matched population, except for preeclampsia, where there was no difference detected between the rates of both populations. Additionally, it is noteworthy that natural abortion was only 1.1 times more likely in alleged violence cases, contrary to findings in the literature suggesting that IPV victims are 1.6 times more likely to experience miscarriage. [16] However, our results are consistent with studies indicating that women with abusive partners are twice as likely to have had an induced abortion. This correlation can be attributed to the increased probability of unintended pregnancies among these women. [61]

### Prevalence ratios vs incidence ratios

When comparing the incidence rates and ratios of health conditions between the suspected and matched populations, we observed a substantial increase for the alleged victims compared to the matched population. This is in contrast to the previous study, which showed higher rates but not as high ratios when compared to the matched population. Upon comparing our incidence rate ratios with the prevalence rate ratios from previous studies on the same population, utilizing information obtained from codes and clinical notes, we noted several discrepancies. [8]

Regarding health risk behaviours, the incidence ratios for alcohol abuse and current smoking are higher (5 and 1.9 times, respectively) than the prevalence ratios (3.3 and 1.4 times). However, for drug abuse, both the incidence and prevalence ratios are 5 times greater in the suspected population in comparison with the matched population.

Traumatic injuries and intoxications showed higher incidence ratios than prevalence ratios for all outcomes except poisoning, where the ratios remained the same.

Contrary to expectations, the incidence ratios for the following mental health disorders were lower than the prevalence ratios: suicidal ideation (5 vs 7.3), social deprivation (4 vs 5), eating disorders (2 vs 2.5), post-traumatic stress disorder (2 vs 8), and antipsychotics use (2.7 vs 2.9). However, for most mental health outcomes, the incidence ratios were higher than the prevalence ratios.

General health and cardiovascular conditions displayed a consistent pattern, showing higher incidence ratios. Significant differences were observed between ratios of the following conditions: hemorrhagic stroke and early heart disease (over 3 times higher in incidence ratio compared to prevalence ratio), chronic kidney disease (with incidence ratio almost 3 times as high as prevalence ratio); and myocardial infarction, ischemic stroke and hypertension (twice the incidence ratio compared to the prevalence ratio).

Regarding gynaecological and obstetric conditions, we observed that the incidence ratios were lower than the prevalence ratios across all outcomes, including inflammatory pelvic disease, which was contrary to our expectations.

On the other hand, respiratory, urinary, inflammatory, and cancer conditions all exhibited higher rates of incidence rations than prevalence ratios: chronic obstructive pulmonary disease (6 vs 1.7), asthma (2.1 vs 1.3), urinary tract infection (1.7 vs 1.4), chronic immune-inflammatory disorder (2 vs 1.3), cervical cancer (2.5 vs 2) and cancer (2.3 vs 1.5)

Our initial hypothesis, suggesting that a significant portion of allegedly victimized individuals may discontinue regular care and become disconnected from the healthcare system, is supported by the observation of considerably higher incidence rates of health conditions among women recorded as alleged victims of IPV compared to previous prevalence estimates. However, we were unable to definitively determine the cause of the loss of follow-up. One potential reason could be the treatment dropout, as women may be reluctant to report their exposure to violence due to concerns about their safety, social marginalization, lack of knowledge, and cultural stigmas. [5,25,26,63–65]

Notably, the most reported health conditions in our study are severe, which may prompt greater attention from healthcare professionals. Thus, we also hypothesize that another potential reason for loss of follow-up could be mortality, particularly considering that the outcomes are more severe and the median age of our population is 41 years, indicating an older demographic where natural deaths may occur.

Additionally, it is crucial to acknowledge that mortality in this population might be influenced by factors beyond natural deaths, including the possibility of homicide given the greater risk of violence-related fatalities among individuals with a history of IPV. [66]

### Limitations and further research

It is essential to acknowledge the limitations of our study and the need for further research in this area. One important limitation is the potential underreporting of the health consequences of IPV, particularly for less severe or indolent outcomes. Victims often hesitate to disclose their experiences, which can also contribute to this underestimation of incidence. It is crucial to recognize the importance of addressing all health consequences, as even seemingly minor symptoms can be indicative of underlying violence. [48] Healthcare professionals should receive training and maintain a high level of suspicion for IPV and violence in general, to ensure appropriate identification and intervention. [7]

It would be relevant to extend this study to other ULS, looking for possible differences among different regions of the country. Additionally, studies aimed at understanding underlying reasons for loss of follow-up among individuals allegedly victimized by IPV are crucial. Prospective studies, tracking participants over time and allowing for the assessment of time-to-event outcomes, can provide valuable insights into the factors contributing to discontinuation of care, whether due to mortality or actual loss of follow-up due to abandonment of care. These studies can also explore the effectiveness of interventions aimed at mitigating loss of follow-up and improving continuity of care for IPV-affected individuals.

## Conclusions

Based on the obtained results, several key conclusions can be drawn:

1. The average age of alleged female victims of IPV was 41 years, with around 75% of the victims being 30 years or older.
2. Poverty and unspecified social problems were higher in the suspected population.
3. Compared to the matched population, women allegedly victims of IPV had higher incidence rates of:
  a) Health risk behaviours: alcohol abuse (5 times higher) and current smoking (almost twice more likely);
  b) Traumatic injuries and intoxications: dislocations, burns, and crushing injuries (3.2, 2.7, and 2.3 times higher, respectively);
  c) Mental health disorders: suicidal ideation (5 times more likely), social deprivation and memory disorders (both 4 times more likely), and sleep disorders (3.1 times more likely);
  d) Medication consumption: antipsychotics, sedatives, antidepressants, anxiolytics (2.7, 2.4, 2.3, and 2 times more likely, respectively);
  e) Physical disorders (except for preeclampsia): hemorrhagic stroke (6.7 times higher), early heart disease and chronic obstructive pulmonary disease (6 times higher), chronic kidney disease and myocardial infarction (both 5 times higher), ischemic stroke (4.5 times higher), and type 2 diabetes (3.9 times higher).
4. Incidence ratios, when compared to prevalence ratios from previous similar populations, were:
  a) Higher for alcohol abuse and current smoking;
  b) Higher for dislocations, crushing injuries, burns, open wounds, fractures, and superficial injuries except for poisoning;
  c) Lower for suicidal ideation, social deprivation, eating disorders, post-traumatic stress disorder, and antipsychotics use;
  d) 3 times higher for hemorrhagic stroke and early heart disease; double the prevalence ratio for myocardial infarction, ischemic stroke and hypertension;
  e) Lower across all gynaecological and obstetric conditions;
  f) Higher for respiratory, urinary, inflammatory and neoplastic conditions.

In conclusion, our study highlights the significant increase in incidence rates of various health conditions among those allegedly victimized compared to the matched population. It provides novel insights by comparing incidence rates with previous prevalence estimates, revealing higher incidence ratios across the majority of health conditions among allegedly female IPV victims. This suggests a potential loss of follow-up among these individuals, possibly due to treatment dropout or mortality, highlighting the urgent need for targeted interventions and enhanced healthcare support for IPV victims.

## Institutional review board statement

Data access for analysis was provided after obtaining approval from the Ethics Committee and the data protection officer of the LHUM—approval codes No. 72/CES/JAS of 16 September 2022 and No. 85/CLPSI/2022 of 5 January 2023, respectively.

## Informed consent statement

Considering that this is a study of databases with an eligible population numbering in the hundreds of thousands, the application of informed consent is not feasible.

## References

[1] United Nations. Convention on the Elimination of All Forms of Discrimination against Women. https://www.un.org/womenwatch/daw/cedaw/. Accessed 2023 December 14. 12346612

[2] Violence against women prevalence estimates, 2018: global, regional and national prevalence estimates for intimate partner violence against women and global and regional prevalence estimates for non-partner sexual violence against women. Geneva: World Health Organization. 2021.

[3] World Bank. Violence against women and girls - what the data tell us. https://genderdata.worldbank.org/data-stories/overview-of-gender-based-violence/. 2022. Accessed 2023 December 14.

[4] Vieira-Pinto P, Muñoz-Barús JI, Taveira-Gomes T, Vidal-Alves MJ, Magalhães T. Intimate partner violence against women. Does violence decrease after the entry of the alleged offender into the criminal justice system?. Forensic Sci Res. 2022;7(1):53–60. doi: 10.1080/20961790.2021.1960616 35341122 PMC8942538

[5] Alves MJV, Manita C, Caldas IM, Fernández-Martinez E, Gomes da Silva A, Magalhães T. Evolution and analysis of cultural and cognitive factors related with domestic violence against women. J Interpers Violence. 2019;34(3):621–41. doi: 10.1177/0886260516645570 27139222

[6] Tjaden P, Thoennes N. Extent, nature, and consequences of intimate partner violence findings from the national violence against women survey. 2000. http://www.ojp.usdoj.gov

[7] Martins H, Assunção L, Caldas IM, Magalhães T. Victims of intimate partner violence. The Physician’s Intervention in the Portuguese National Health Service. J Fam Viol. 2014;29(3):315–22. doi: 10.1007/s10896-014-9586-5

[8] Clemente-Teixeira M, Magalhães T, Barrocas J, Dinis-Oliveira RJ, Taveira-Gomes T. Health outcomes in women victims of intimate partner violence: A 20-year real-world study. Int J Environ Res Public Health. 2022;19(24):17035. doi: 10.3390/ijerph192417035 36554916 PMC9779804

[9] Wong J, Mellor D. Intimate partner violence and women’s health and wellbeing: impacts, risk factors and responses. Contemp Nurse. 2014;46(2):170–9. doi: 10.5172/conu.2014.46.2.170 24787250

[10] Rivara F, Adhia A, Lyons V, Massey A, Mills B, Morgan E, et al. The effects of violence on health. Health Aff (Millwood). 2019;38(10):1622–9. doi: 10.1377/hlthaff.2019.00480 31589529

[11] Novais M, Henriques T, Vidal-Alves MJ, Magalhães T. When problems only get bigger: The impact of adverse childhood experience on adult health. Front Psychol. 2021;12:693420. doi: 10.3389/fpsyg.2021.693420 34335410 PMC8318698

[12] Campbell JC. Health consequences of intimate partner violence. Lancet. 2002;359(9314):1331–6. doi: 10.1016/S0140-6736(02)08336-8 11965295

[13] Breiding MJ, Black MC, Ryan GW. Chronic disease and health risk behaviors associated with intimate partner violence-18 U.S. states/territories, 2005. Ann Epidemiol. 2008;18(7):538–44. doi: 10.1016/j.annepidem.2008.02.005 18495490

[14] Wright EN, Hanlon A, Lozano A, Teitelman AM. The impact of intimate partner violence, depressive symptoms, alcohol dependence, and perceived stress on 30-year cardiovascular disease risk among young adult women: A multiple mediation analysis. Prev Med. 2019;121:47–54. doi: 10.1016/j.ypmed.2019.01.016 30695719

[15] World Health Organization. Violence against women. https://www.who.int/news-room/fact-sheets/detail/violence-against-women. 2021. Accessed 2023 December 14.

[16] García-Moreno C, World Health Organization. Global and regional estimates of violence against women: prevalence and health effects of intimate partner violence and non-partner sexual violence. 2013.

[17] Sarkar NN. The impact of intimate partner violence on women’s reproductive health and pregnancy outcome. J Obstet Gynaecol. 2008;28(3):266–71. doi: 10.1080/01443610802042415 18569465

[18] Jackson KT, Marshall C, Yates J. Health-related maternal decision-making among perinatal women in the context of intimate partner violence: A scoping review. Trauma Violence Abuse. 2023;25(3):1899–910. doi: 10.1177/15248380231198876 37728102 PMC11155210

[19] Chisholm CA, Bullock L, Ferguson JEJ 2nd. Intimate partner violence and pregnancy: epidemiology and impact. Am J Obstet Gynecol. 2017;217(2):141–4. doi: 10.1016/j.ajog.2017.05.042 28551446

[20] Ibrahim ZM, Ahmed WAS, El-Hamid SA, Hagras AM. Intimate partner violence among Egyptian pregnant women: incidence, risk factors, and adverse maternal and fetal outcomes. Clin Exp Obstet Gynecol. 2015;42(2):212–9. doi: 10.12891/ceog1829.2015 26054122

[21] Pico-Alfonso MA, Garcia-Linares MI, Celda-Navarro N, Blasco-Ros C, Echeburúa E, Martinez M. The impact of physical, psychological, and sexual intimate male partner violence on women’s mental health: depressive symptoms, posttraumatic stress disorder, state anxiety, and suicide. J Womens Health (Larchmt). 2006;15(5):599–611. doi: 10.1089/jwh.2006.15.599 16796487

[22] Saberi E, Eather N, Pascoe S, McFadzean M-L, Doran F, Hutchinson M. Ready, willing and able? A survey of clinicians’ perceptions about domestic violence screening in a regional hospital emergency department. Australas Emerg Nurs J. 2017;20(2):82–6. doi: 10.1016/j.aenj.2017.02.001 28279677

[23] Sardinha L, Maheu-Giroux M, Stöckl H, Meyer SR, García-Moreno C. Global, regional, and national prevalence estimates of physical or sexual, or both, intimate partner violence against women in 2018. Lancet. 2022;399(10327):803–13. doi: 10.1016/S0140-6736(21)02664-7 35182472 PMC8885817

[24] Devries KM, Mak JY, Bacchus LJ, Child JC, Falder G, Petzold M, et al. Intimate partner violence and incident depressive symptoms and suicide attempts: a systematic review of longitudinal studies. PLoS Med. 2013;10(5):e1001439. doi: 10.1371/journal.pmed.1001439 23671407 PMC3646718

[25] Meyer S. Why women stay: A theoretical examination of rational choice and moral reasoning in the context of intimate partner violence. Aust N Zeal J Criminol. 2012;45(2):179–93. doi: 10.1177/0004865812443677

[26] Sani AI, Pereira D. Mothers as victims of intimate partner violence: The decision to leave or stay and resilience-oriented intervention. Soc Sci. 2020;9(10):174. doi: 10.3390/socsci9100174

[27] McCloskey LA, Lichter E, Williams C, Gerber M, Wittenberg E, Ganz M. Assessing intimate partner violence in health care settings leads to women’s receipt of interventions and improved health. Public Health Rep. 2006;121(4):435–44. doi: 10.1177/003335490612100412 16827445 PMC1525344

[28] Ross J, Walther V, Epstein I. Screening risks for intimate partner violence and primary care settings: implications for future abuse. Soc Work Health Care. 2004;38(4):1–23. doi: 10.1300/J010v38n04_01 15149903

[29] Tiwari A, Yuk H, Pang P, Fong DYT, Yuen F, Humphreys J, et al. Telephone intervention to improve the mental health of community-dwelling women abused by their intimate partners: a randomised controlled trial. Hong Kong Med J. 2012;18(Suppl 6):14–7. 23249846

[30] McFarlane JM, Groff JY, O’Brien JA, Watson K. Secondary prevention of intimate partner violence: a randomized controlled trial. Nurs Res. 2006;55(1):52–61. doi: 10.1097/00006199-200601000-00007 16439929

[31] Gibbs A, Jewkes R, Willan S, Washington L. Associations between poverty, mental health and substance use, gender power, and intimate partner violence amongst young (18-30) women and men in urban informal settlements in South Africa: A cross-sectional study and structural equation model. PLoS One. 2018;13(10):e0204956. doi: 10.1371/journal.pone.0204956 30281677 PMC6169941

[32] Ahmadabadi Z, Najman JM, Williams GM, Clavarino AM. Income, gender, and forms of intimate partner violence. J Interpers Violence. 2020;35(23–24):5500–25. doi: 10.1177/0886260517719541 29294851

[33] Yonfa EDA, Fasol M, Cueva CM, Zavgorodniaya AC. Intimate partner violence: A literature review. Open Psychol J. 2021;14(1):11–6. doi: 10.2174/1874350102114010011

[34] Giacomini SG, Machado MM, de Santana OM, Rocha SG, de Aquino CM, Gomes LG, et al. Intimate partner violence among women living in families with children under the poverty line and its association with common mental disorders during COVID-19 pandemics in Ceará, Brazil. BMC Public Health. 2023;23(1):1299. doi: 10.1186/s12889-023-16233-2 37415137 PMC10327360

[35] Gillum TL. The intersection of intimate partner violence and poverty in Black communities. Aggress Violent Behav. 2019;46:37–44. doi: 10.1016/j.avb.2019.01.008

[36] Laksono AD, Wulandari RD, Matahari R, Suharmiati. Socioeconomic differences of intimate partner violence among married women in Indonesia: Does poverty matter?. Indian J Community Med. 2023;48(2):304–9. doi: 10.4103/ijcm.ijcm_254_22 37323739 PMC10263050

[37] Ince-Yenilmez M. The role of socioeconomic factors on women’s risk of being exposed to intimate partner violence. J Interpers Violence. 2022;37(9–10):NP6084–111. doi: 10.1177/0886260520966668 33047645

[38] Rempel E, Donelle L, Hall J, Rodger S. Intimate partner violence: a review of online interventions. Inform Health Soc Care. 2019;44(2):204–19. doi: 10.1080/17538157.2018.1433675 29537928

[39] El-Bassel N, Gilbert L, Wu E, Go H, Hill J. Relationship between drug abuse and intimate partner violence: a longitudinal study among women receiving methadone. Am J Public Health. 2005;95(3):465–70. doi: 10.2105/AJPH.2003.023200 15727978 PMC1449203

[40] Clark CJ, Everson-Rose SA, Alonso A, Spencer RA, Brady SS, Resnick MD, et al. Effect of partner violence in adolescence and young adulthood on blood pressure and incident hypertension. PLoS One. 2014;9(3):e92204. doi: 10.1371/journal.pone.0092204 24658452 PMC3962399

[41] Yalch MM, Christodoulou J, Rotheram-Borus MJ, Tomlinson M. Longitudinal association between intimate partner violence and alcohol use in a population cohort of South African women. J Interpers Violence. 2023;38(1–2):NP1718–37. doi: 10.1177/08862605221092068 35473455

[42] Carbone-López K, Kruttschnitt C, Macmillan R. Patterns of intimate partner violence and their associations with physical health, psychological distress, and substance use. Public Health Rep. 2006;121(4):382–92. doi: 10.1177/003335490612100406 16827439 PMC1525352

[43] McNutt LA, Carlson BE, Rose IM, Robinson DA. Partner violence intervention in the busy primary care environment. Am J Prev Med. 2002;22(2):84–91. doi: 10.1016/s0749-3797(01)00407-x 11818176

[44] Krug EG, Dahlberg LL, Mercy JA, Zwi AB, Lozano R. World report on violence and health. 2002. 10.1016/S0140-6736(02)11133-0 12384003

[45] Bhatt B, Bhatt N, Karki A, Giri G, Baaniya B, Neupane B, et al. Intimate partner violence against married women of reproductive age in Nepal during the COVID-19 pandemic. Heliyon. 2023;9(9):e20117. doi: 10.1016/j.heliyon.2023.e20117 37809852 PMC10559861

[46] Boyle A, Jones P, Lloyd S. The association between domestic violence and self harm in emergency medicine patients. Emerg Med J. 2006;23(8):604–7. doi: 10.1136/emj.2005.031260 16858090 PMC2564159

[47] Jónasdóttir D, Thorsteinsdottir T, Ásgeirsdóttir TL, Lund SH, Arnarsson EÖ, Ashikali E, et al. Women and intimate partner violence: Prevalence of hospital visits and nature of injuries in the Icelandic population. Scand J Public Health. 2021;49(3):260–7. doi: 10.1177/1403494820916093 32308135

[48] Chen Z, Ma W, Li Y, Guo W, Wang S, Zhang W, et al. Using machine learning to estimate the incidence rate of intimate partner violence. Sci Rep. 2023;13(1):5533. doi: 10.1038/s41598-023-31846-8 37015976 PMC10073277

[49] Devries K, Watts C, Yoshihama M, Kiss L, Schraiber LB, Deyessa N, et al. Violence against women is strongly associated with suicide attempts: evidence from the WHO multi-country study on women’s health and domestic violence against women. Soc Sci Med. 2011;73(1):79–86. doi: 10.1016/j.socscimed.2011.05.006 21676510

[50] Ellsberg M, Jansen HAFM, Heise L, Watts CH, Garcia-Moreno C, WHO Multi-country Study on Women’s Health and Domestic Violence against Women Study Team. Intimate partner violence and women’s physical and mental health in the WHO multi-country study on women’s health and domestic violence: an observational study. Lancet. 2008;371(9619):1165–72. doi: 10.1016/S0140-6736(08)60522-X 18395577

[51] Coston BM. Disability, sexual orientation, and the mental health outcomes of intimate partner violence: A comparative study of women in the U.S. Disabil Health J. 2019;12(2):164–70. doi: 10.1016/j.dhjo.2018.11.002 30448101

[52] Stene LE, Dyb G, Jacobsen GW, Schei B. Psychotropic drug use among women exposed to intimate partner violence: A population-based study. Scand J Public Health. 2010;38(5 Suppl):88–95. doi: 10.1177/1403494810382815 21062843

[53] Wuest J, Merritt-Gray M, Ford-Gilboe M, Lent B, Varcoe C, Campbell JC. Chronic pain in women survivors of intimate partner violence. J Pain. 2008;9(11):1049–57. doi: 10.1016/j.jpain.2008.06.009 18701353

[54] Tho Nhi T, Hanh NTT, Hinh ND, Toan NV, Gammeltoft T, Rasch V, et al. Intimate partner violence among pregnant women and postpartum depression in Vietnam: A longitudinal study. Biomed Res Int. 2019;2019:4717485. doi: 10.1155/2019/4717485 31179324 PMC6507254

[55] Samelius L, Wijma B, Wingren G, Wijma K. Somatization in abused women. J Womens Health (Larchmt). 2007;16(6):909–18. doi: 10.1089/jwh.2006.0103 17678462

[56] Mendonça MFS de, Ludermir AB. Intimate partner violence and incidence of common mental disorder. Rev Saude Publica. 2017;51:32. doi: 10.1590/S1518-8787.2017051006912 28423141 PMC5396502

[57] Chandan JS, Thomas T, Bradbury-Jones C, Taylor J, Bandyopadhyay S, Nirantharakumar K. Risk of cardiometabolic disease and all-cause mortality in female survivors of domestic abuse. J Am Heart Assoc. 2020;9(4):e014580. doi: 10.1161/JAHA.119.014580 32063124 PMC7070197

[58] Dong M, Giles WH, Felitti VJ, Dube SR, Williams JE, Chapman DP, et al. Insights into causal pathways for ischemic heart disease: adverse childhood experiences study. Circulation. 2004;110(13):1761–6. doi: 10.1161/01.CIR.0000143074.54995.7F 15381652

[59] Goldberg X, Espelt C, Porta-Casteràs D, Palao D, Nadal R, Armario A. Non-communicable diseases among women survivors of intimate partner violence: Critical review from a chronic stress framework. Neurosci Biobehav Rev. 2021;128:720–34. doi: 10.1016/j.neubiorev.2021.06.045 34252471

[60] Hess KL, Javanbakht M, Brown JM, Weiss RE, Hsu P, Gorbach PM. Intimate partner violence and sexually transmitted infections among young adult women. Sex Transm Dis. 2012;39(5):366–71. doi: 10.1097/OLQ.0b013e3182478fa5 22504601 PMC3856434

[61] Silverman JG, Raj A. Intimate partner violence and reproductive coercion: global barriers to women’s reproductive control. PLoS Med. 2014;11(9):e1001723. doi: 10.1371/journal.pmed.1001723 25226396 PMC4165751

[62] Imayilova L, Macro I. Intimate Partner Violence and Unintended Pregnancy in Azerbaijan, Moldova, and Ukraine. 2010. www.measuredhs.com

[63] Juarros-Basterretxea J, Fernández-Álvarez N, Torres-Vallejos J, Herrero J. Perceived reportability of intimate partner violence against women to the police and help-seeking: A national survey. Psychosoc Interv. 2024;33(1):55–64. doi: 10.5093/pi2024a3 38298215 PMC10826975

[64] Kimberg L, Vasquez JA, Sun J, Anderson E, Ferguson C, Arreguin M, et al. Fears of disclosure and misconceptions regarding domestic violence reporting amongst patients in two US emergency departments. PLoS One. 2021;16(12):e0260467. doi: 10.1371/journal.pone.0260467 34855809 PMC8638952

[65] Ingram M, McClelland DJ, Martin J, Caballero MF, Mayorga MT, Gillespie K. Experiences of immigrant women who self-petition under the Violence Against Women Act. Violence Against Women. 2010;16(8):858–80. doi: 10.1177/1077801210376889 20679184

[66] Pereira AR, Vieira DN, Magalhães T. Fatal intimate partner violence against women in Portugal: a forensic medical national study. J Forensic Leg Med. 2013;20(8):1099–107. doi: 10.1016/j.jflm.2013.09.015 24237830

